# First Draft Genome Sequence of a Pearl Millet Blast Pathogen, Magnaporthe grisea Strain PMg_Dl, Obtained Using PacBio Single-Molecule Real-Time and Illumina NextSeq 500 Sequencing

**DOI:** 10.1128/MRA.01499-18

**Published:** 2019-05-16

**Authors:** Ganesan Prakash, Aundy Kumar, Neelam Sheoran, Rashmi Aggarwal, Chellapilla Tara Satyavathi, Surendra K. Chikara, Arpita Ghosh, Rakesh Kumar Jain

**Affiliations:** a Division of Plant Pathology, ICAR-Indian Agricultural Research Institute, New Delhi, India; b ICAR-All India Coordinated Research Project on Pearl Millet, Jodhpur, Rajasthan, India; c Eurofins Genomics India Private Limited, Bengaluru, Karnataka, India; University of Maryland School of Medicine

## Abstract

The first draft genome sequence of the pearl millet blast pathogen Magnaporthe grisea PMg_Dl from India is presented. The genome information of *M. grisea* will be useful to understand the *Magnaporthe* speciation, genetic diversity, environmental adaptation, and pathogenic and host range determinants.

## ANNOUNCEMENT

In India, rice (Oryza sativa L.), pearl millet [Pennisetum glaucum (L.) R. Br.], and finger millet (Eleusine coracana L.) are among the cereals affected by blast disease incited by *Magnaporthe* species (1, 2). Pearl millet blast is caused by Magnaporthe grisea. Although *Magnaporthe* genomes infecting various cereals have been sequenced by several laboratories, the genome sequence of a pearl millet-infecting *Magnaporthe* sp. is unavailable so far (3–5). Here, we report for the first time the draft genome sequence of Magnaporthe grisea strain PMg_Dl, which infects pearl millet from India. A single spore colony of Magnaporthe grisea strain PMg_Dl was isolated from an infected leaf of pearl millet cultivar ICMB95444 in the experimental farm at the Indian Council of Agricultural Research (ICAR)-Indian Agriculture Research Institute in New Delhi, India, by the lesion print technique/modified spore drop method (6). Strain PMg_Dl is also pathogenic to other hosts, such as wheat, oat, and barley, under experimental conditions, but it was nonpathogenic to rice and finger millet.

A monosporidial culture of strain PMg_Dl was grown at 28°C with constant shaking (150 rpm) in potato dextrose broth (HiMedia, Mumbai, India) for 7 to 10 days. The high-quality genomic DNA was extracted from fungal mycelia using cetyltrimethylammonium bromide (CTAB) and the phenol-chloroform method, followed by RNase A treatment. The quality and quantity were assessed spectrophotometrically as well as by a Qubit fluorometer and 0.8% agarose gel electrophoresis. Genome sequencing of PMg_Dl was performed using the Illumina NextSeq 500 platform with an average read length of 2 × 150 bp and the PacBio RS II platform with P6-C4 chemistry. The mate pair (MP) sequencing library was prepared using an Illumina Nextera MP sample preparation kit using the gel-free protocol, and the shotgun (whole-genome sequencing [WGS]) paired-end (PE) sequencing library was prepared using a TruSeq nano DNA library prep kit. High-molecular-weight DNA was used to prepare one SMRTbell library of 5 to 8 kb for sequencing on the PacBio platform using the hairpin adaptor protocol for ultra-long-read sequencing. Total data generated were 13.1-Gb PE reads (number of reads, 43,962,401), 3.4-Gb mate-paired reads (number of reads, 17,160,010), and 1.1-Gb PacBio reads (number of reads, 148,768). The PE raw reads were filtered using Trimmomatic (7), and the MP reads were filtered using NextClip (8). The high-quality PE, MP, and PacBio reads of the PMg_Dl sample were assembled into scaffolds by SPAdes (9) genome assembler version 3.12.0, and scaffolding was carried out by SSAKE-based scaffolding of preassembled contigs after extension (SSPACE) version 3.0 (10).

In total, the assembly of 341 scaffolds resulted in a genome size of 47.90 Mb with an *N*_50_ value of 765,468 bp. The G+C content of the genome is 47.3%. A total of 10,451 genes in PMg_Dl were predicted using Magnaporthe grisea as a gene prediction model (11). BLASTX with an E value of 1e−5 resulted in the annotation of 9,649 coding DNA sequences (CDSs) that had significant BLAST hits. The majority of hits were found to be against Magnaporthe oryzae 70-15. Gene ontology (GO) annotations of the genes were carried out using Blast2GO to classify the function of the predicted genes (12). A total of 3,151 genes were annotated into 24 functional categories based on the KEGG pathway database via KEGG Automatic Annotation Server (KAAS) (13). Genes involved in pathogenicity, virulence, and effectors were annotated using pathogen-host interaction database (PHI-base) version 4.5 (14). A total of 849 CDSs involved in pathogenicity, virulence, and effector genes were annotated (12, 14). The data presented here will be useful for further studies on the genetic and functional characterization of Magnaporthe grisea, which infects pearl millet and other cereals.

### Data availability.

The raw data were submitted to the SRA under accession numbers SRR8573217, SRR8573216, and SRR8776454. The SRA BioProject accession number is PRJNA494483. This whole-genome shotgun project has been deposited at DDBJ/ENA/GenBank under the accession number RHLM00000000. The version described in this paper is the first version, RHLM01000000.
